# Global public health implications of human exposure to viral contaminated water

**DOI:** 10.3389/fmicb.2022.981896

**Published:** 2022-08-30

**Authors:** Adedayo Ayodeji Lanrewaju, Abimbola Motunrayo Enitan-Folami, Saheed Sabiu, Joshua Nosa Edokpayi, Feroz Mahomed Swalaha

**Affiliations:** ^1^Department of Biotechnology and Food Science, Durban University of Technology, Durban, South Africa; ^2^Water and Environmental Management Research Group, Engineering and Agriculture, University of Venda, Thohoyandou, South Africa

**Keywords:** enteric viruses, gastroenteritis, outbreak, wastewater, wastewater-based epidemiology

## Abstract

Enteric viruses are common waterborne pathogens found in environmental water bodies contaminated with either raw or partially treated sewage discharge. Examples of these viruses include adenovirus, rotavirus, noroviruses, and other caliciviruses and enteroviruses like coxsackievirus and polioviruses. They have been linked with gastroenteritis, while some enteric viruses have also been implicated in more severe infections such as encephalitis, meningitis, hepatitis (hepatitis A and E viruses), cancer (polyomavirus), and myocarditis (enteroviruses). Therefore, this review presents information on the occurrence of enteric viruses of public health importance, diseases associated with human exposure to enteric viruses, assessment of their presence in contaminated water, and their removal in water and wastewater sources. In order to prevent illnesses associated with human exposure to viral contaminated water, we suggest the regular viral monitoring of treated wastewater before discharging it into the environment. Furthermore, we highlight the need for more research to focus on the development of more holistic disinfection methods that will inactivate waterborne viruses in municipal wastewater discharges, as this is highly needed to curtail the public health effects of human exposure to contaminated water. Moreover, such a method must be devoid of disinfection by-products that have mutagenic and carcinogenic potential.

## Introduction

The world is increasingly faced with daunting challenges in meeting the expanding requirements for clean water as the existing supplies of freshwater are in short supply owing to (i) prolonged droughts; (ii) population growth; (iii) strict health-based guidelines; and (iv) contending demands from diverse users (Mancosu et al., 2015). Due to these facts and many others, water protection against possible biological and chemical contaminants is becoming critical in water resources management. Enteric viruses are one of the emerging biological contaminants responsible for the sporadic outbreak of waterborne illnesses worldwide (La Rosa et al., 2012; Upfold et al., 2021). Compared with bacteria and protozoa, viruses are smaller, measuring between 20 and 350 nm in diameter (Health Canada, 2019). Viruses are obligate intracellular parasites containing bundles of gene strands of either RNA or DNA as a core nucleic acid surrounded by a protective coat called protein capsid (Clemente et al., 2012). Sometimes, the capsid protein is enclosed in an additional spikey envelope. In an enveloped virus, the nucleocapsid is bounded by a lipid bilayer which results from the modified host cell membrane with an outer layer of virus envelope glycoproteins (Zwart et al., 2009; Lázaro et al., 2018). Unlike bacterial cells, which are free-living entities, viruses employ the host cell environment to multiply. Studies have shown that viruses can latch onto host cells, as seen in several outbreaks including the current coronavirus (COVID-19) pandemic caused by the severe acute respiratory syndrome coronavirus 2 (SARS-CoV-2) (Shang et al., 2020).

According to Clemente et al. (2012), patients suffering from gastroenteritis may defecate about 10^5^–10^11^ virus particles per gram of stool which in turn gain entrance into environmental media through the discharge of contaminated wastewater (Wu et al., 2020; Yeo et al., 2020). Though enteric viruses are the smallest enteric pathogens with a low infective dose, they are robust and difficult to detect and inactivate (Gonzales-Gustavson et al., 2019; Farkas et al., 2020a) hence, they serve as a route of potential health risk at a low exposure dose (Zwart et al., 2009; Zhu et al., 2018). Thus, the transmission of human enteric viruses *via* the water route is becoming more widely recognized as a potential cause of human diseases such as gastroenteritis, encephalitis, meningitis, and hepatitis, among others (World Health Organization, 2014; Tang et al., 2020).

There are several cases where viruses are detected in water deemed compliant with bacterial indicators, and the consumption of such water has been reported to result in waterborne diseases (Umesha et al., 2008; Chen et al., 2021). Even when subjected to conventional wastewater treatments, only about 20–80% of the enteric viruses get inactivated due to their resistance to chemical disinfectants (Jiang et al., 2001; La Rosa et al., 2012), thus leading to the discharge of viruses into the aquatic environment. They can survive unfavorable conditions such as temperature and pH among others for an extended period in environmental waters owing to their small size (Bertrand et al., 2012; Meixell et al., 2013), inertness, and the presence of viral capsid.

Several studies have detected enteric viruses in seawater, surface and groundwater, municipal wastewater influent, and inadequately treated wastewater effluent (Saxena et al., 2015; Shih et al., 2017; Hendriksen et al., 2019; Pang et al., 2019; Verani et al., 2019; Shaheen et al., 2020; Tang et al., 2020; Farkas et al., 2020a). However, their occurrence is not limited to those water matrices, but they have also been found in drinking water systems and recreational water (Umesha et al., 2008; Upfold et al., 2021). Another route of enteric virus transmission is food products contaminated with irrigation water (Benko et al., 2002; Bouseettine et al., 2020). They could be transmitted *via* food, such as shellfish grown in contaminated water, wastewater irrigated farm produce, open defecation, untreated contaminated surface water, and exposure to inadequately treated wastewater due to poor infrastructure (Umesha et al., 2008; Haramoto et al., 2018). Therefore, this review focuses on the occurrence of enteric viruses in water and wastewater sources, their public health implications, and associated diseases due to human exposure to waterborne enteric viruses. Finally, we examined how the identified public health concerns can be minimized.

## Enteric viruses of public health importance

**Figure 1:**
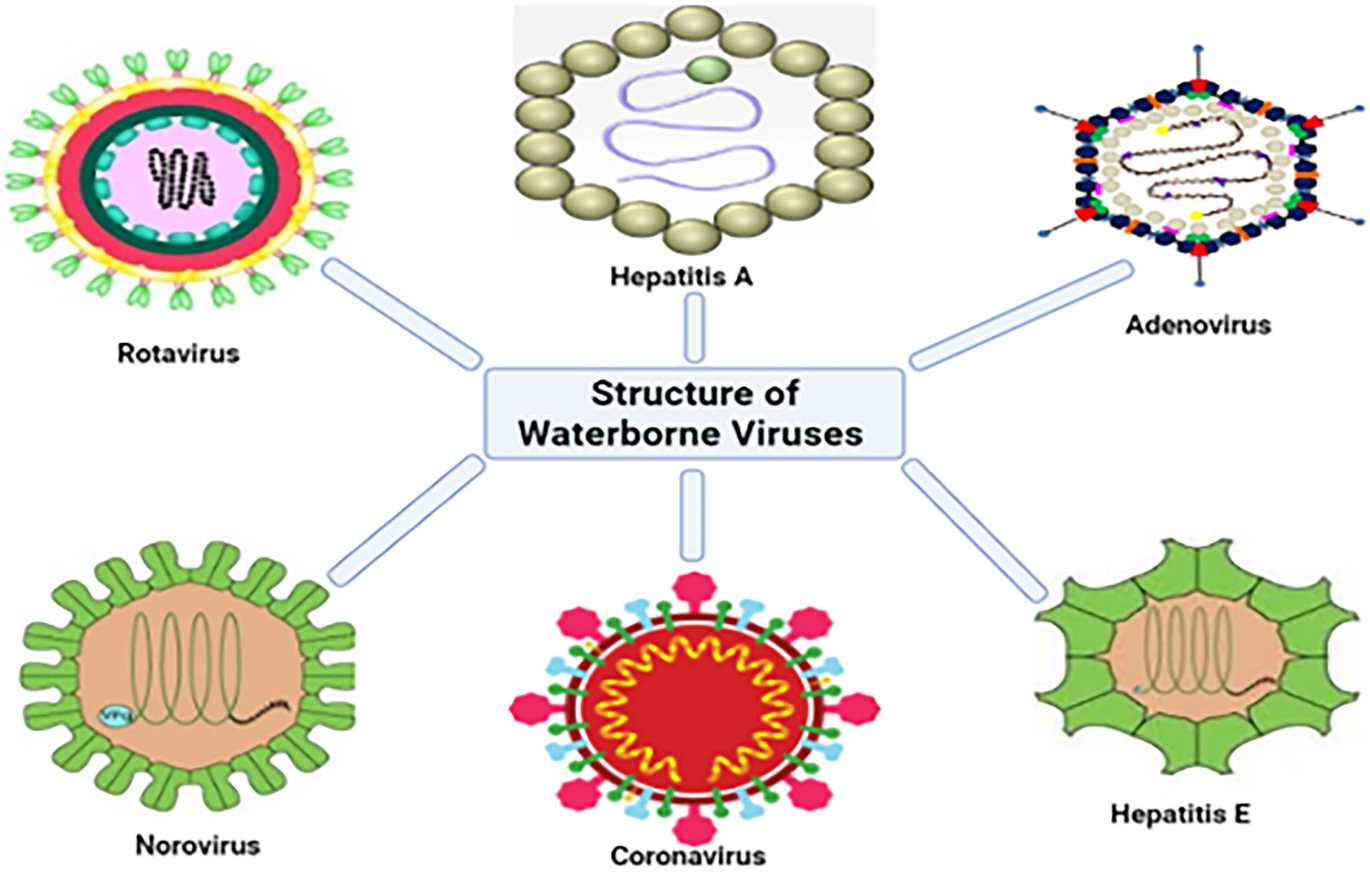
Structures of some of the viruses that have been detected in water sources (adapted from Altintas et al., 2015).

Worldwide, more than 150 enteric viruses are associated with waterborne diseases. The prevalence of Hepatitis A Virus (HAV), Adenovirus (AdV), Rotaviruses (RV) and Enteroviruses (EVs), Astroviruses (AstVs), Noroviruses (NoVs), and bacteriophages in surface waters (Radin, 2014), dams and treated drinking water (Fernandez-Cassi et al., 2018; Opere, 2019), treated effluent from wastewater treatment plants (WWTPs; Gonzales-Gustavson et al., 2019) and the detection in post chlorinated water is not exempted (La Rosa et al., 2012). Figure 1 shows both enveloped (e.g., coronaviruses) and non-enveloped viruses found in treated and untreated wastewater. In the recent SARS-CoV-2 outbreak, scientists discovered that the virus responsible for the pandemic; SARS-CoV-2 is shed in feces, detected in the sewerage system, and treated effluent (Ahmed et al., 2020; Haramoto et al., 2020; Randazzo et al., 2020). Furthermore, NoV, AdV, AstV, EV, HAV, RV, and hepatitis E virus (HEV) are transmitted *via* water (Gibson, 2014; Bouseettine et al., 2020).

### Adenovirus

Human adenoviruses (HAdV) are icosahedral capsid structures, non-segmented, non-enveloped, and double-stranded DNA viruses with a diameter between 90 and 100 nm (Opere, 2019). The family Adenoviridae is divided into five genera: Genus Mastadenovirus, which infects mammals; Aviadenovirus, which infects birds; Siadenovirus, which infects birds and frogs and Atadenovirus, which infects a wide variety of hosts such as aves, reptiles, and marsupial hosts (Arnold and MacMahon, 2017) and lastly, the newly proposed genera, Ichtadenovirus (Benko et al., 2002). Human adenovirus (HAdV) is a nonlinear envelope single-stranded DNA in the Adenoviridae family and genus Mastadenoviruses (Crenshaw et al., 2019). Within the Mastadenovirus genus, there are presently 103 HAdV serotypes, categorized into seven species (A–G) (Kosulin, 2019; Mennechet et al., 2019).

Many adenoviruses are shed in high amounts with more than 10^11^ particles per gram of faeces, urine, or respiratory excretions (Hewitt et al., 2013). Across the world, these viruses have been found in wastewater, both influent and effluent (Mena and Gerba, 2008; Iaconelli et al., 2017a). Specifically, many HAdV species have been detected in surface water (Sibanda and Okoh, 2012; Opere, 2019) and wastewater (Osuolale and Okoh, 2015; Iaconelli et al., 2017b). Generally, they can survive as extra circular chromosomes or through integration into the DNA of the host (Fu et al., 2019).

In general, HAdVs are prevalent etiological agents of intestinal, respiratory, and ophthalmic diseases. Several clinical features are caused in the respiratory tract, eyes, gastrointestinal tract, and other organs due to the initial propagation, which may occur in the mucosa of the pharynx, conjunctiva, or intestinal mucosa (Howley and Lowy, 2007). Symptoms of infection include gastroenteritis, upper and lower respiratory tract infection, conjunctivitis, pneumonia, myocarditis, and encephalitis (Russell, 2009; Akello et al., 2020). They are generally mild and self-limiting, but in immunocompromised individuals, they can evolve into severe infections with unique manifestations (Khanal et al., 2018). In both developed and developing nations, the occurrence of enteric HAdVs in childhood diarrhea range from 1 to 8% and 2 to 31%, respectively (Meqdam and Thwiny, 2007; Hassou et al., 2020). Between 2007 and 2019, HAdV was found in about 10.8% of all gastroenteritis incidents among children below five in Sub-Saharan Africa (Oppong et al., 2020).

### Rotavirus

Rotavirus (RV), belonging to the Reoviridae family, is a relevant and significant waterborne pathogen (Atabakhsh et al., 2021). The genus comprises five species (A–E), two tentative species (F and G) as well as an unassigned species (ADRV-N) which is referred to as RVH (Desselberger, 2014). The rotavirus genome has 11 segments of dsRNA with a size that ranges from 0.6 to 33 kilobase pairs (Bouseettine et al., 2020). Segmentation in the RV genome allows the rearrangement mechanism and production of new strains with different combinations of genome segments resulting in genetic diversity, boosting the evolution, and emerging new strains of RVs each year (Esona et al., 2010; Menezes et al., 2020).

In humans, serogroups A–C cause gastroenteritis, while group A causes severe diarrhea in young children (Bouseettine et al., 2020). Group A is the most common cause of gastrointestinal disease in children below the age of 5, with severe outcomes such as hospitalization and death (Prez et al., 2015; Magana-Arachchi and Wanigatunge, 2020). The principal agents of infectious dehydrating diarrhea in infants are the rotavirus group A [RVA], which is linked to most human RV infections. RVA genotypes G1 P[8], G2 P[4], G3 P[8], and G9 P[8] are associated with most human gastroenteritis and easily undergo genetic recombination (Leite et al., 2008; Tate et al., 2010). Wa-like (G1-P[8]-I1-R1-C1- M1-A1-N1- T1-E1-H1) and DS-1-like (G2-P[4]-I2-R2-C2-M2-A2- N2-T2-E2-H2) are the two main genogroups identified from the analyses of the complete genome sequences of human rotavirus (HRV) strains to contain the majority of wild-type HRV strains (Tate et al., 2010). The G1P [8] strain is the most predominant in Europe, with about 69.4% of infections in America and some Asian countries (Fongaro et al., 2015). However, there has been a wide level of reduction in the cases of severe childhood diarrhea in countries where routine childhood vaccination against rotavirus has been entrenched. In the same vein, there seems to be a reduction in the number of unvaccinated children due to the protection already offered to vaccinated children, thereby suggesting herd protection because of the vaccination (Fongaro et al., 2015).

Rotavirus infection has been linked to about 258 million cases of diarrhea in children under 5 years worldwide (Troeger et al., 2018). Nevertheless, RV continues to be the leading cause of annual childhood deaths associated with diarrhea worldwide despite the effective introduction of vaccines in more than 106 countries (Kim et al., 2021). The virus has been estimated to be responsible for 122,000–215,000 diarrheic child deaths between 2013 and 2017 annually (Tate et al., 2016; Troeger et al., 2018). In a study conducted to investigate the link between the contaminated water source and rotaviral infection among 184 children under 5 years with acute watery diarrhea, the highest rotaviral infection was observed in children that consume contaminated pond water (94.44%). It was followed by children that were not breastfed exclusively (83.87%) and 64.00% in children living in areas with lower socio-economic conditions (Habib et al., 2021).

### Enterovirus

Enterovirus (EV) is a spherical non-enveloped virus containing a 7,500-nucleotide positive-sense single-stranded RNA genome. They are 7.3–7.4 kb in length and are protected by an icosahedral capsid (Opere, 2019). Enteroviruses belong to the family Picornaviridae and are extremely small, with diameters ranging from 22 to 30 nm (Magana-Arachchi and Wanigatunge, 2020). The genus enterovirus has more than 300 known serotypes and 15 species (Chen et al., 2020). The different species include polioviruses, coxsackieviruses A and B, enteroviruses, and echoviruses and they have been linked with human infections (Table 1; Bouseettine et al., 2020).

**Table 1:** Species of human enteroviruses and associated pathologies (adapted from Bouseettine et al., 2020).

Species	Serotypes	Associated pathologies
Enteroviruses	68–71	Encephalitis, conjunctivitis, meningitis, and paralysis
Echovirus	1–9, 11–21, 24–27, 29–34	Encephalitis, conjunctivitis, meningitis, paralysis, and gastroenteritis
Coxsackievirus A	1–22, 24	Encephalitis, fever, meningitis, and paralysis
Coxsackievirus B	1–6	Encephalitis, gastroenteritis, myalgia, meningitis, paralysis, and pericarditis
Poliovirus	1–3	Encephalitis, gastroenteritis, and pericarditis

Millions of people worldwide have been infected with enteroviruses, which have caused the irregular outbreak of illnesses in both advanced and evolving nations (Lugo and Krogstad, 2016; Cassidy et al., 2018; Smuts et al., 2018; Puenpa et al., 2019). Of the seven species to which human EVs belong, only four species (A–D) infect the gastrointestinal tract (Baggen et al., 2018). Enterovirus 71 (EV-A71) is a human enteric virus linked with hand-foot-and-mouth disease (HFMD), while EV-D68 is associated with acute flaccid paralysis (Lee and Chi, 2014; Baggen et al., 2018). A total of 2,967 cases of EV and Parechovirus infections were documented in the United States of America between 2014 and 2016. The most frequently reported enterovirus is the EV-D68 which is responsible for 68% of identified types in 2014 and accounted for 56% of all documented types between 2014 and 2016. Other enteroviruses that were also frequently reported include echovirus 30 (13.1%), coxsackievirus A6 (12.5%), echovirus 18 (9.5%), and coxsackievirus B3 (9%) (Abedi et al., 2018).

The mode of transmission of enterovirus is the faecal-oral route through different sources, such as contaminated water, food, or person-to-person contact. The faecal-oral route becomes problematic when there is exposure to aerosols that are transmitted from any surface or groundwater sources (Gao et al., 2012). Enteroviruses are robust organisms as they can survive critical changes in both pH and temperature (Okoh et al., 2010). Therefore, they have been proposed as a criterion for assessing viral contamination of environmental waters because they are common and shed for an extended period in the environment (La Rosa et al., 2010a). Studies that focused on their removal at WWTPs revealed that they are more resistant to treatment when compared to some other enteric viruses such as adenoviruses and noroviruses (La Rosa et al., 2010b). Several studies have identified them in both raw and treated sewage (La Rosa et al., 2010b; Simmons et al., 2011).

### Norovirus

Noroviruses, which were initially referred to as Norwalk or Norwalk-like viruses, are small non-enveloped single-stranded RNA viruses of 27–38 nm in diameter (Rani et al., 2021). They are members of the family Caliciviridae (Upfold et al., 2021). Norwalk virus is composed of a single strand of RNA and bounded by many copies of a single protein organized into a protective capsid (Wang et al., 2018b). According to Thorne and Goodfellow (2014), it has been frequently revealed that Norovirus is widespread and that infections occur more in the human population. Noroviruses are classified into 10 different genogroups (GI–GX; Laconi et al., 2020). Generally, NoVs GI, GII, and GIV infect man, while GI is less found in patients with acute gastroenteritis across the globe than in GII strains (Koopmans et al., 2002). The most common genogroup in man is the three distinct clusters in GII to which the porcine NoVs belong. This has raised public health concerns regarding the potential NoVs recombinant strains in causing zoonotic transmission (Shen et al., 2012; Okada et al., 2019; Laconi et al., 2020). Generally, gastroenteritis linked with NoVs is self-limiting, although young children, the elderly, and immunocompromised individuals could experience long-term symptoms (Trivedi et al., 2013; Petrignani et al., 2018). Norovirus GI and GII are majorly responsible for human infections; however, for about 10 years now, the genogroup GII.4 has been linked with most human NoVs diseases (Siebenga et al., 2009; Vinjé, 2015; Mans et al., 2016). A novel strain of genotype GII.17 (GII.P17-GII.17) was reported in Asia to be responsible for gastroenteritis linked with NoV in the year 2014 and has subsequently been documented worldwide (Chan et al., 2015, 2017; Lu et al., 2015; Hoa-Tran et al., 2017; Zhou et al., 2019).

The transmission of noroviruses is primarily *via* the faecal-oral route. It transpires through the intake of contaminated water or by direct contact with persons already infected and on environmental surfaces (Zhou et al., 2016; Boonchan et al., 2017; Kittigul et al., 2019). Drinking water and recreational water polluted from sewage are the sources of several outbreaks of NoVs (Kittigul et al., 2019; Prado et al., 2019; Tryfinopoulou et al., 2019). According to de Graaf et al. (2016), GI variants are more linked with transmission through the water route than variant GII.4, which relates to person–person contact. The GI strains of Norovirus are believed to be stable in water compared to the GII strains because they are more likely to be transmitted through water than other transmission routes. Across the globe, NoVs have been detected in different water bodies, including sewages, rivers, recreational waters, municipal water, and groundwater (La Rosa et al., 2007, 2008, 2010a; Miura et al., 2019).

### Astrovirus

Human astroviruses (HAstVs) are the third most frequent cause of gastroenteritis, and they were first detected in 1975 (Vu et al., 2017). They are small with sizes ranging from 28 to 30 nm and are non-enveloped viruses having single-stranded RNA (+ssRNA) genomes with about 6,400–7,900 nucleotides. Genera Mamastrovirus (MAstV) and Avastrovirus (AAstV) make up the family Astroviridae (Appleton, 1975; De Benedictis et al., 2011; Donato and Vijaykrishna, 2017). Human astroviruses (HAstVs) are a vital cause of gastroenteritis in infants and young children (De Benedictis et al., 2011). They are found in surface and groundwaters meant for drinking purposes, marine waters, and wastewater effluents (Magana-Arachchi and Wanigatunge, 2020). A study on the molecular detection of gastroenteritis viruses reported that HAstV had a lesser occurrence than EV, RV, NoV, and AdV (Chitambar et al., 2012). The aged and individuals with weak immune system are susceptible to gastroenteritis linked with HAstV (Vu et al., 2017; Wohlgemuth et al., 2019). This could be credited to the pathogenic role of the virus being performed in immunocompromised individuals. In addition, HAstVs have been linked to central nervous system (CNS) infections including encephalitis and acute flaccid paralysis in people with compromised immunity (Cordey et al., 2016; Vu et al., 2016). It could also be due to the inability of the individuals to mount an active inflammatory response to the virus hence a reflection of their highly immunocompromised state.

### Hepatitis A virus

Hepatitis A virus (HAV), which is a member of the Hepatovirus genus belonging to the family Picornaviridae, is non-enveloped with an icosahedral structure of an average of 30 nm in diameter and a naked RNA genome (Smith and Simmonds, 2018; Bouseettine et al., 2020). The capsid contains a densely packed icosahedral configuration of 60 protomers, each of which includes three polypeptides VP1, VP2, and VP3 (McKnight and Lemon, 2018). Studies have reported that about 1.5 million people are infected with HAV annually, which is underestimated due to the asymptomatic presentation of the virus and limited epidemiologic information on the virus (World Health Organization, 2017b; Lemon et al., 2018).

As the major causative agent of non-parenteral hepatitis in developing nations as well as endemic infections in developing countries, the primary mechanism of transmission of the HAV is the faecal-oral route and direct contact with an infected person (Mehta and Reddivari, 2021). The outbreaks of HAV infection are mostly associated with water supplies (Jothikumar et al., 2005). The consumption of raw or improperly cooked oysters and clams from sewage-contaminated water has led to many outbreaks of HAV infection (Elbashir et al., 2018). Fever, anorexia, malaise, abdominal discomfort, nausea, and jaundice are some of the numerous symptoms that equally result from HAV infection and could to liver damage (La Rosa et al., 2012).

Hepatitis A virus is found in the feces and urine of diseased individuals through which it contaminates the soil and water. The virus is detected in water and wastewater globally; however, an area’s sanitary conditions determine the virus’s prevalence (World Health Organization, 2012c). People living in areas with poor sanitation infrastructure are more prone to contracting the virus, especially among children (World Health Organization, 2012b). According to Rodriguez-Lazaro et al. (2012), HAV can subsist for about 60 days in tap water, while it can stay alive for more than 6 weeks in river water, can stay above 8 weeks in groundwater, and for about 30 weeks in seawater. The virus has been found in different water environments such as raw wastewaters (Osuolale and Okoh, 2015), treated effluents (Van Zyl et al., 2019), surface waters (Marie and Lin, 2017; Shaheen et al., 2019), and drinking waters (Moreno et al., 2009).

### Hepatitis E virus

Hepatitis E virus (HEV) is a member of the family Hepeviridae and belongs to the Orthohepevirus genus and the Orthohepevirus A species. HEV is a non-enveloped, positive sense, ssRNA virus (Denner, 2019) that causes significant acute hepatitis worldwide thereby resulting in sporadic infections and epidemics (Raji et al., 2021). The infection is usually asymptomatic in the general population (Khuroo and Kamili, 2003). The virus is regarded as zoonotic with swine and other animals such as rabbits serving as the reservoir for human infections (Ruggeri et al., 2013; Syed et al., 2018). Hepatitis E virus is another waterborne pathogen threatening global health in developing and developed countries (Raji et al., 2021). Currently, eight HEV genotypes have been identified (Upfold et al., 2021), and genotypes 1–4 and 7 are known to be the main threats to humans. They are transmitted through the ingestion of contaminated water, causing acute hepatitis (Purdy et al., 2017).

Ruggeri et al. (2013), posited that HEV strains that belong to genotypes 1 and 2 are the cause of the most sporadic outbreak of hepatitis in developing countries of Africa, Asia, and Mexico (Smith and Simmonds, 2018). Genotypes 3, 4, and 7 are basically associated with zoonotic transmission (Marek et al., 2010) and are generally connected with irregular and grouped infections in advanced countries (Nimgaonkar et al., 2018). It is transmitted *via* the faecal-oral pathway and spreads readily through water bodies polluted with human faeces. Globally, the virus has been detected in different water bodies including rivers (Iaconelli et al., 2015), and raw wastewater (Iaconelli et al., 2017a; Wang et al., 2018a), among others.

### Aichivirus

Aichivirus (AiV) belongs to the Kobuvirus genus and the family Picornaviridae (Rivadulla and Romalde, 2020). It is one of the significant causative organisms of gastroenteritis in humans and is transmitted *via* the faecal-oral route from contaminated food or water (Yamashita et al., 2000; Adams et al., 2013; Kebe et al., 2021). Aichivirus consists of Human AiV (HAiV) 1, Murine Kobuvirus (MuKV) 1, and Canine Kobuvirus (CaKV) 1. Human AiV is composed of three genotypes (A–C; Yamashita et al., 2000; Shaheen et al., 2020; Kebe et al., 2021), and the infection rate of AiV in gastroenteritis cases worldwide is low between 0.4 and 6.5% (Rivadulla and Romalde, 2020; Taghinejad et al., 2020; Upfold et al., 2021). However, specific antibodies produced against the virus are found in about 80–90% of adults (Kitajima and Gerba, 2015; Rivadulla and Romalde, 2020), which is a pointer to the asymptomatic condition of most infections (Bergallo et al., 2017). Furthermore, this virus is often detected in connection with other enteric pathogens (Oh et al., 2006; Ambert-Balay et al., 2008; Kaikkonen et al., 2010; Japhet et al., 2019). Diarrhea, abdominal pain, vomiting, and fever are some clinical signs and symptoms of human AiV virus infection (Yamashita et al., 2000). Aichivirus has been reported in Africa, Asia, South America, and Europe (Oh et al., 2006; Lodder et al., 2013). The virus is normally passed out in the faeces of humans directly or after discharge of treated or untreated sewage (Lodder et al., 2013; Kebe et al., 2021), and can be adopted as a potential indicator of wastewater reclamation system (Kitajima et al., 2014; Farkas et al., 2020b).

### Coronaviruses

Coronaviruses (CoVs) are positive-sense single-stranded RNA viruses (Qu et al., 2020; Yeo et al., 2020) with more than 30 different species and have the biggest genome of RNA viruses of 30 kb (Amirian, 2020; Amoah et al., 2020). Coronaviruses are classified into four types: Alphacoronavirus (Alpha-CoV), Betacoronavirus (Beta-CoV), Gammacoronavirus (Gamma-CoV), and Deltacoronavirus (Delta-CoV; Delta-CoV; Qu et al., 2020). The coronavirus virion is typically spherical, with a diameter of 60–140 nm, and is enclosed by an outer viral envelope covered by projections (9–12 nm; Zhu et al., 2020). CoVs were thought to be insignificant human pathogens until the beginning of this century. The latest outbreak in Wuhan, China toward the end of 2019 was the emergence of a novel coronavirus (Lu et al., 2020). It was initially referred to as HCoV-19 but later officially named Severe Acute Respiratory Syndrome Coronavirus 2 (SARS-CoV-2); because the novel virus is most closely linked to the SARS-CoV virus that was implicated in the 2003 SARS outbreak (Lu et al., 2020; World Health Organization, 2020).

The SARS-CoV-2 is responsible for a variety of common cold-like and acute respiratory diseases (Qu et al., 2020; Yeo et al., 2020). COVID-19 is the respiratory disease induced by SARS-CoV-2 (World Health Organization, 2020), with clinical symptoms including diarrhea, nausea, fever, cough, rhinorrhea, dyspnea, or severe pneumonia and myalgia (Guan et al., 2020; Liu et al., 2020; Yeo et al., 2020). Nevertheless, a considerable proportion of individuals remain symptomless despite testing positive for SARS-CoV-2 (Bai et al., 2020; Lai et al., 2020; Liu et al., 2020; Rothe et al., 2020). Evidence from the present COVID-19 outbreak proposes that about 2–35% of patients develop gastrointestinal (GI) symptoms such as diarrhea, abdominal discomfort, and vomiting; however, this is less common than respiratory symptoms (Yeo et al., 2020; Wang et al., 2020a), and this has resulted in the presence of the virus in feces and sewage (Amirian, 2020; Pan et al., 2020). Therefore, this virus has been detected in untreated wastewater and rivers (La Rosa et al., 2020; Medema et al., 2020; Rimoldi et al., 2020; Sherchan et al., 2020; Pillay et al., 2021), as well as in treated wastewaters (Haramoto et al., 2020; Randazzo et al., 2020).

## Prevalence of enteric viruses in freshwater and wastewater sources

Critical tools for detection and prevention of the further spread of diseases and outbreaks include clinical surveillance and monitoring of waterborne pathogens. However, disease prevalence is under-reported through clinical testing because it is typically restricted to those who are ill to the extent of seeking treatment and testing (Cacciò and Chalmers, 2016). Hence, there is a lag indicator for predicting an outbreak in a community. Therefore, there is a desperate need for cost-effective and improved monitoring methods to detect numerous waterborne diseases in a community, preferably in real-time. To this end, wastewater-based epidemiology (WBE) has recently garnered much attention as an early warning technique for a range of waterborne infectious diseases (Zahedi et al., 2021).

Wastewater-based epidemiology (WBE) has been employed before now to monitor drug abuse within a community (Castiglioni et al., 2006) and other chemical pollutants (Choi et al., 2018), as well as the prevalence of poliovirus (Pöyry et al., 1988; Berchenko et al., 2017). Broadly, it entails the detection of nucleic acids or other biomarkers excreted in feces and urine in wastewater to offer detailed health information about a community (Mao et al., 2020). The approaches encompass the detection of pathogens released in saliva, sputum, mucus, vomitus, and phlegm that are frequently trapped in wastewater (Zahedi et al., 2021). Accordingly, WBE is thus equal to the collection and analysis of a large community-based combined sample of faeces, saliva, vomitus, sputum, urine, shed skin, and other substances released during personal cleansing, washing, bathing, and excretion. Hence, it provides a sensitive technique for tracking temporal alterations and variety in pathogen concentrations within a community (Xagoraraki and O’Brien, 2020). Another benefit of the direct analysis of wastewater samples is the presence of higher populations of pathogens in wastewater compared to environments where inadequately treated wastewater is discharged (Zahedi et al., 2021).

Wastewater passes through a series of treatment processes, including oxidation ponds, coagulation, activated sludge, chlorination, and ozonation until good effluent quality is achieved. However, about 50–90% of the waterborne viruses can only be removed *via* different treatment processes, thereby releasing a significantly high viral load into the environment that humans become exposed to with accompanying public health diseases (Gersberg et al., 1988; Zhu et al., 2005; Charles et al., 2008; Okoh et al., 2010; La Rosa et al., 2012; Sano et al., 2016). Supplementary Table 1 shows the prevalence of selected waterborne enteric viruses across different countries in various water environments, which portends the potential risk of the transmission of the virus. The employment of bacterial indicator species to assess the quantity of the virus load in wastewater effluents is one of such limitations. This has been faulted as an inefficient technique for monitoring the quality of wastewater (Gersberg et al., 1988; Hunter, 1997; Zhu et al., 2005; Bofill-Mas et al., 2006; Charles et al., 2008; Karmakar et al., 2008; Okoh et al., 2010; Farkas et al., 2020b).

The prevalence of HAdV in both 60 stool samples from children with acute gastroenteritis and 96 sewage samples collected from the Zenin wastewater treatment plant was investigated within a community in Egypt from January to December 2017 (Elmahdy et al., 2019). The virus was detected in 17 (28.3%) of stool, 27 (84.4%) of raw sewage, 16 (50%) of treated sewage, and 25 (78%) of sludge samples throughout a whole year of sample collection. According to Elmahdy et al. (2019), the occurrence of HAdV in the treated effluent of the WWTP portends a serious public health problem. Similarly, the prevalence of AdVs in treated wastewater in Brazil was reported by Quintão et al. (2021). The study reported the occurrence of AdVs in 27.2% (61/224) of the investigated samples. It was observed that the occurrence of the virus was higher in downstream samples than that in upstream samples. In another study, F species HAdVs serotype 41 (79.2%) and C species PAdVs serotype 5 (18.1%) were higher than other serotypes in the water samples collected from Puzi River, Taiwan. On the other hand, the prevalence of NoV GII was more than GI in the same river. Specifically, GII.4 (21.2%) and GII.17 (18.2%) were reported as the predominant genotypes. The occurrence of both AdVs and NoVs was higher in the winter compared to the spring, summer, and autumn seasons.

Furthermore, Iaconelli et al. (2017a), reported the prevalence of EV, AdV, HAV, HEV, and NoV in raw and treated wastewater. Adenovirus was the most frequently detected virus in raw wastewater (81%) compared to 33% in treated effluents, followed by EV, with 13 samples detected in raw sewage and 3 samples in treated effluents. Norovirus GI or GII were detected in 10 raw wastewater and 4 treated effluents. Likewise, HEV was detected in one sewage sample, while HAV was 33% in raw sewage and 19% in treated effluents (Iaconelli et al., 2017a). Conversely, Hepatitis A and E virus outbreaks were linked to contaminated drinking water in 72% (109/151) and 49 (38%) of the 128 outbreaks (Kumar et al., 2015). Also, Miao et al. (2018), assessed the prevalence of EV, RV, AstV, NoV GII, and AdV in Jinhe River, China. The detection frequency differs as follows; 91.7% for AdV, 81.3% for NoV GII, 79.2% for EV and AstV, and 70.8% for RV. In addition, the authors reported a seasonal pattern concerning the prevalence of the detected viruses in which there was an abundance of EVs in summer whereas RVs, AstVs, NoV GII, and AdVs showed opposite seasonal trends.

The prevalence of different astrovirus strains in different water matrices such as wastewater in the US, groundwater, and river in Nepal has also been reported (Hata et al., 2018). The identified strains include types 6 and 7 classical human astroviruses, emerging type 5 VA-astroviruses, and putative recombinants. The prevalence of classical and VA-astroviruses was reported during the cooler months while it was during the warmer months that MLB-astroviruses were detected (Hata et al., 2018). Aichivirus was found in Nepal in different water sources such as sewage pipes, rivers, groundwater, and a house that received its water from a tanker (Haramoto and Kitajima, 2017). Variations in AiV detection with a significant prevalence of AiV B were reported in the study. When compared to shallow tube wells, where AiV was detected in 1 out of 15 samples (7%), the frequency of AiV detection was substantially higher in shallow dug wells, where it was discovered in 10 out of 22 samples (45%). This variation could be attributed to the fragile structure of dug wells often composed of stone or brick compared to that of tube wells. Conversely, AiV was detected in 50% of river water samples examined in a study conducted in Iran (Azhdar et al., 2019). According to a survey carried out by Moreira and Bondelind (2017), *Cryptosporidium*, norovirus, *Giardia*, *Campylobacter*, and rotavirus were the waterborne pathogens responsible for drinking waterborne outbreaks during 2000–2014. Authors further reported that contamination of surface water sources affected most consumers which led to gastrointestinal diseases, while the distribution network was responsible for most individual incidents (Moreira and Bondelind, 2017).

Also, rotaviruses in surface water and wastewater have been reported in different parts of the world, especially in developing countries (Taylor et al., 2001; Verheyen et al., 2009; Rezaeinejad et al., 2014). Nevertheless, they are less often detected in environmental samples compared to AdVs (Spilki et al., 2013). The distribution of RV infection is seasonal, with the incidence reaching the peak during winter (Suzuki et al., 2005). However, it has been reported all year round worldwide (Osuolale and Okoh, 2016). The RV strains are linked with diseases like acute diarrhea in humans and animals (Almeida et al., 2018; Menezes et al., 2020). The transmission of rotavirus is majorly *via* the ingestion of food and water that has been contaminated with human wastes. In most cases, newborns or young children with gastroenteritis are infected with subclinical illness from an older sibling or mother. The shedding of the virus from the intestinal tract occurs before diarrhea sets in or even after it has been reported (Mukhopadhya et al., 2013).

During the global COVID-19 outbreak in Japan, Hata et al. (2021) investigated the presence of SARS-CoV-2 RNA in wastewater samples and the number of confirmed COVID-19 cases in the study area was compared. During the study period, a total of 45 influent samples were collected from five wastewater treatment plants in Ishikawa and Toyama regions in Japan. The virus was detected in 21 out of the 45 influent samples. There was an increase in the frequency of detection as the total number of confirmed cases in 100,000 people surpassed 10 in each region; however, SARS-CoV-2 RNA could still be detected even when there was a reduction in the number of confirmed cases. This could be attributed to the continuous shedding of the virus from discharged asymptomatic individuals. Hata et al. (2021), opined that the viral monitoring of wastewater could be adopted as an early warning signal of COVID-19 outbreaks in Japan.

Considering the occurrence of these viruses in water bodies, there is a need to carefully monitor treated wastewater before discharge; however, the employed methods have some drawbacks. The number of viruses contained in environmental media can vary significantly; however, it is a function of the type of sample being examined. High viral concentration could be detected and quantified in wastewater or sludge from treatment plants using a very small sample volume (Haramoto et al., 2018). Nevertheless, a significantly small volume which could be less than 1 ml that will be concentrated from the wastewater sample is required for the downstream detection experiments. Ultrafiltration, adsorption/elution, flocculation, and ultracentrifugation, among other techniques, have been utilized to concentrate viruses from wastewater (Prata et al., 2012; Haramoto et al., 2018). A dependable and appropriate method of concentration should have a capacity for high viral recovery, repeatable results, be suitable for detecting a wide array of viruses, create a minimal volume of viral concentrate, be rapid and cost-effective to operate (Bosch, 1998).

Unfortunately, all these criteria have not been found in any single technique for the concentration of viruses in water. This has made the concentration of viruses in water considerably more difficult. Hence, rapid action is necessary to track viruses in fresh and wastewater sources. Even though no strict standards have been set in addressing the concentration of viruses in treated wastewater effluents before disposal. Their elimination before discharge into environmental media is critical to minimize potential outbreaks and accompanying diseases due to exposure to humans (Haramoto et al., 2018). It is crucial to optimize and evaluate the available concentration techniques to improve virus recovery and build a highly effective process that will go a long way in combating virus outbreaks in the future hence, research should be geared toward achieving this goal (Ibrahim et al., 2021).

## Public health implications of waterborne enteric viral diseases

The genomic content and capsid proteins of enteric viruses differ, nevertheless, they share some similar attributes making them more of public health threat regarding the risk of drinking contaminated water. The non-enveloped virus can remain active in water bodies for extended periods (Reynolds et al., 2008). Considering those attributes, drinking inadequately disinfected water contaminated with faeces could lead to waterborne disease outbreaks (Gall et al., 2015a; Sano et al., 2016; Adelodun et al., 2021). It is also noteworthy that viruses could be transmitted through three major routes; ingestion, inhalation, and direct contact *via* interaction with skin and eyes (swimming) leading to respiratory and ocular infections (Gall et al., 2015a).

Usually, waterborne enteric viruses are associated with gastrointestinal diseases, epidemics, and acute hepatitis. Adenovirus, AstVs, HAV, HEV, RVs, NoVs, and other caliciviruses, and enteroviruses, including coxsackieviruses and polioviruses are classified by WHO as waterborne viral pathogens with modest to high health importance (WHO, 2017c). These viruses are linked with gastroenteritis and diarrhea coupled with the severity of other symptoms such as fever and abdominal cramps among others. Table 2 shows the different waterborne enteric viruses and related diseases. Gastroenteritis is the most frequent pathology connected with enteric virus’ infections (Bányai et al., 2018). Various gastroenteritis outbreaks have been associated with drinking sewage-contaminated water containing some of these enteric viruses which include AdVs, EVs, NoVs, RVs, AstVs, AiVs among others (Maunula et al., 2009; Räsänen et al., 2010; Kauppinen et al., 2019). For instance, a minimum of 33 outbreaks were connected to drinking contaminated water between 2009 and 2014 in the United States of America (Control and Prevention, 2013). The most important cause of gastrointestinal illness across the world is human noroviruses. Due to increased environmental robustness, human noroviruses genogroup I is usually involved in waterborne cases (Matthews et al., 2012), while genogroup II with the large majority of cases is transmitted from person to person and presumed to be connected with food (Ahmed et al., 2014).

**Table 2:** Enteric viruses and related diseases.

Enteric viruses	Related diseases	References
Adenoviruses	Gastroenteritis, respiratory disease, and conjunctivitis	Gibson, 2014; Kumar et al., 2014; La Rosa et al., 2012
Enteroviruses	Gastroenteritis, meningitis, myocarditis, respiratory disease, encephalitis, and conjunctivitis	Bouseettine et al., 2020; Gibson, 2014; Kumar et al., 2014; La Rosa et al., 2012
Poliovirus	Poliomyelitis, meningitis, and encephalitis	Bouseettine et al., 2020; Kumar et al., 2014
Coxsackievirus	Meningitis, encephalitis, paralysis, and myocarditis	Bouseettine et al., 2020; Kumar et al., 2014
Astroviruses	Gastroenteritis	Gibson, 2014; Kumar et al., 2014
Hepatitis viruses A, E	Hepatitis	Gibson, 2014; Kumar et al., 2014
Noroviruses	Gastroenteritis	Gibson, 2014; Kumar et al., 2014
Sapoviruses	Gastroenteritis	Kumar et al., 2014
Rotavirus	Gastroenteritis	Gibson, 2014; Kumar et al., 2014
Aichivirus	Gastroenteritis	Bouseettine et al., 2020
Coronavirus	Gastroenteritis and respiratory disease	Dongdem et al., 2009; La Rosa et al., 2012

Likewise, diarrhea has also emerged as the world’s second-biggest cause of death in children under 5 years (Murray and Newby, 2012). It is one of the major recurrent waterborne infections, with 1.7 billion cases recorded each year (WHO, 2017a), and 525,000 children’s deaths (Pooi and Ng, 2018). According to the global burden of disease investigated in 2015, 1.2 million deaths and 71.7 million disability-adjusted life years (DALYs) coupled with 1.1 million deaths and 61.1 million DALYs from diarrheal diseases all resulted from contaminated water sources (Forouzanfar et al., 2016). The primary cause of diarrhea contracted by drinking contaminated water is bacteria; however, little or no attention is being paid to viral pathogens in water sources as well as their impact on public health. Gibney et al. (2017), conducted a study in 2010 on the disease burden of cryptosporidiosis, campylobacteriosis, giardiasis, nontyphoidal salmonellosis, and norovirus ascribed to the waterborne transmission of selected enteric pathogens in Australia. Most waterborne disease cases were linked to norovirus, a waterborne enteric virus (479,632; 95% confidence interval [UI]: 0–1,111,874), followed by giardiasis and campylobacteriosis. Based on a global study of groundwater-related enteric illness outbreaks survey, an alarming rise in groundwater-related acute gastrointestinal infections (AGI) was reported between 1948 and 2015, with 649 incidents identified (Murphy et al., 2017).

Recently, Carol et al. (2021) investigated the causative agent responsible for an outbreak of acute gastroenteritis (AGE) among 174 pupils that were involved in a school trip between 30 January and 3 February 2017 at a holiday camp in Catalonia. The authors discovered about 41 episodes of AGE with symptoms ranging from abdominal pain (73.8%), nausea (64.3%), vomiting (54.8%), diarrhea (45.2%), and headache (42.9%). The outbreak also was linked to the consumption of NoV GII contaminated water samples (crude RR: 1.72, 95% CI: 1.01–2.92; adjusted RR: 1.88, 95% CI 1.03–3.56). According to the World Health Organization/United Nations Children Emergency Fund, (2015) reports, untreated water is used by 663 million people worldwide. Although, individuals living in poverty-stricken or rural areas and developing regions are affected by a lack of access to safe drinking water. Notwithstanding, even people living in developed countries with advanced water and wastewater treatment facilities are not spared from waterborne diseases (Adelodun et al., 2021). Furthermore, it has been estimated that by the year 2030, approximately 1.6 billion people (19% of the global population) will lack clean water, leading to more waterborne outbreaks and illnesses (World Health Organization/United Nations Children Emergency Fund, 2021).

The Integrated Disease Surveillance Programme (IDSP) reported 804,782 hepatitis cases and 291 outbreaks in India between 2011 and 2013. Hepatitis A testing revealed 44,663 (7.4%) positive cases out of 599,605 total cases, and HEV testing revealed 19,508 (10.4%) positive cases out of 187,040 total cases. Hepatitis E virus accounted for 78 (48%) out of 163 (56%) outbreaks with known etiologies as hepatitis A accounted for 54 (33%), while hepatitis A and E accounted for 19 (12%), (Kumar et al., 2015). In a hepatitis A and E surveillance carried out between 2015 and 2017 in India, 23 disease outbreaks were reported of which 4 outbreaks occurred in 2015, 12 in 2016, and 7 in 2017 (Kadri et al., 2018). Twelve of the total outbreaks were related to hepatitis A infection, 10 to hepatitis E infection, while 1–8 cases of jaundice with no hepatitis A or hepatitis E virus were identified. During the study period, a total of 393 cases of hepatitis A or E were detected. Of the 50 water samples that were examined, 38 were unsuitable for human consumption hence the authors opined that both HAV and HEV outbreaks were due to human exposure to contaminated water (Kadri et al., 2018).

In addition, there was an increase in diarrhea cases reported in Mgcawu District, Northern Cape province, and eThekwini Metropolitan Municipality, KwaZulu-Natal province, South Africa (Shonhiwa et al., 2020). The reported diarrhea outbreaks that affected children under age 5 coincided with the yearly South Africa rotavirus season in 2013 (Shonhiwa et al., 2020). In the same vein, a gastroenteritis outbreak linked to swimming in the lagoon in KwaZulu-Natal Coast, South Africa, resulted in more than 600 cases within 3 weeks in December 2016/January 2017 (Sekwadi et al., 2018). Also, RVs, AstVs, NoVs GI.6, GII.3, and GII.6, were the waterborne viruses identified in the lagoon water samples. The prevalence of the viruses is due to the discharge from WWTPs effluent into the river that feeds the lagoon (Sekwadi et al., 2018). Similarly, in 2017, there was a rotavirus outbreak in India, with a 22.8% attack rate due to drinking water from contaminated wells (Joshi et al., 2019). The Hepatitis E virus was also linked with the largest viral waterborne outbreak in India in which about 80,000 people were affected (Naik et al., 1992; Farkas et al., 2020b).

With above 6 million deaths worldwide as of March 2022, COVID-19, the highly contagious infectious disease caused by SARS-CoV-2, had a devastating impact on the world’s demographics and is emerging as the most significant global health crisis since the period of the influenza pandemic of 1918 (Cascella et al., 2022). The COVID-19 pandemic has resulted in the emergence of significant health challenges owing to its contagious nature and the absence of efficient medical treatment (Coccia, 2021). Respiratory symptoms are the most common in COVID-19 individuals. However, research suggests that gastrointestinal (GI) symptoms such as diarrhea, nausea/vomiting, and abdominal discomfort are common in COVID-19 patients, with a frequency of up to 31.9% (Cholankeril et al., 2020; Remes-Troche et al., 2020). The possibility of the waterborne transmission of SARS-CoV-2 began to draw increasing attention due to the detection of its genetic marker in different water matrices as highlighted earlier. However, there is currently no scientific evidence that the viral RNA is infectious in water and wastewater and can be contacted in water. This gives room for more future studies to look comprehensively into this.

Apart from the loss of lives associated with the unavailability of safe drinking water and poor sanitation, the World Bank estimated an annual economic loss of US$260 billion globally (World Health Organization, 2012a). The infections associated with contaminated water and enteric viral outbreaks are considered under-reported irrespective of the country’s socio-economic condition because the symptoms are generally mild, and people rarely seek medical treatment for self-limiting illnesses (Control and Prevention, 2013). Generally, in healthy individuals, viral infections are self-limiting; however, in children under the age of five, the aged, pregnant women, and immunocompromised patients, greater morbidity could result. Hence, it makes the surveillance of diseases associated with enteric viruses challenging (Cortez et al., 2017; Li et al., 2017). Unfortunately, few broad-spectrum antiviral drugs exist for the treatment of those diseases (Gall et al., 2015a).

## Strategies for the removal of waterborne viruses

Detecting and quantifying the various types of viruses in wastewater are critical for preventing diseases and creating strategic responses to outbreaks. Nevertheless, removing enteric viruses from wastewater is equally essential to avert their spread through food, water, or other pathways. However, the transmission of waterborne viruses is common in developing countries due to their poor sanitation. For instance, there has been a threat to global polio eradication. This is due to the possible movement of indigenous wild poliovirus (WPV) into polio-free countries. Also, it could be through the importation of WPV from polio-endemic countries as well as the release of WPV into the environment from laboratory stocks (Sharma et al., 2015). Hence, every country must detect WPV circulation and other waterborne viruses *via* a sensitive surveillance system.

Although, physical removal of pathogens *via* conventional methods, ultraviolet light or chemical oxidants like chlorine, chloramines, and ozone have been employed, unfortunately, the capacity to resist disinfection due to viral particle size (Ibrahim et al., 2021), and the presence of viral capsid make virus removal a challenging task for most available technologies as well as wastewater epidemiologist. Till date, chlorine disinfectant is being used for water treatment to enhance the deactivation of pathogens and maintain a residual concentration in the distribution line (Crittenden et al., 2012). The availability of chlorine made it a common disinfectant in water purification (Rosario-Ortiz et al., 2016); however, it raises the risk of creating possibly mutagenic and carcinogenic disinfection by-products like bromate and chlorite (Sharma et al., 2014; Gall et al., 2015b) that could pose major health risks to humans (Agency, 2006; Richardson and Postigo, 2011).

Generally, the side effects and limitations associated with the use of conventional techniques for the disinfection of water made it inappropriate in the long run. As highlighted, harmful disinfection by-products are formed when chlorine reacts with natural organic matter found in water sources (Hrudey and Charrois, 2012). The by-products released from the use of those conventional oxidants as disinfectants have been reported to be harmful to human health. Another danger for humans that is associated with the use of conventional disinfectants is their non-specificity as higher doses are needed for disinfection (Kumar et al., 2020). Specifically, studies have shown that the reoviruses may be more sensitive to chlorine disinfection than enteroviruses (Betancourt and Gerba, 2016). Unfortunately, attaining complete sterilization and disinfection has not been confirmed to be achieved by any conventional wastewater treatment procedures (Sano et al., 2016). Hence, finding solutions to reduce this problem has become an issue of keen interest. Therefore, research should be geared toward the development of an effective method of disinfection for the removal of waterborne enteric viruses. In addition, such methods must be devoid of the formation of disinfection by-products which characterizes the chemical methods of disinfection owing to the reaction of residual chlorine with organic matter to form possible mutagenic and carcinogenic by-products.

## Conclusion

This article reviews the public health implications of human exposure to viral contaminated water. The presence of enteric viruses in water comes with accompanying public health implications that cannot be ignored. Although the presence of other microbes in wastewater has been the focus of research before now, the risk of diseases associated with the presence of viruses in water is by far greater than that of other microbes as just an insignificant quantity of viruses is adequate to cause diseases when compared to other microorganisms.

The detection and quantification of the various human viruses in environmental water are critical for public health concerning the prevention of diseases as well as response to outbreaks. Hence, conducting regular viral monitoring of treated wastewater discharged into the environment is important for the prevention of diseases associated with exposure to viral contaminated water. Meanwhile, the presence of viral particles in water and wastewater does not mean that the particles are infectious but are pointers to potential infections and health risk burdens that could emanate from the matrices. Hence, there is an urgent need for further studies to highlight infectious fractions of viral particles disseminated *via* wastewater and water resources for effective risk assessment and epidemiologic purposes. Nevertheless, removing viruses from wastewater using other alternative treatment methods in a bid to stop their transmission *via* the ingestion of contaminated water should be considered. Therefore, a search for a more holistic and cost-effective disinfection method that will inactivate waterborne viruses in water is highly needed to curtail the public health effects of human exposure to contaminated water.

## Author contributions

AL, AE-F, and FS conceived the review idea. AL wrote the first draft. AL, AE-F, SS, and JE reviewed and edited the manuscript. All authors contributed to the article and approved the submitted version.

## Funding

This work was funded by the Water Research Commission (WRC) of South Africa (Project no. K5/C2020-2021-00181). We were also supported by our institution, the Durban University of Technology, South Africa.

## Conflict of interest

The authors declare that the research was conducted in the absence of any commercial or financial relationships that could be construed as a potential conflict of interest.

## Publisher’s note

All claims expressed in this article are solely those of the authors and do not necessarily represent those of their affiliated organizations, or those of the publisher, the editors and the reviewers. Any product that may be evaluated in this article, or claim that may be made by its manufacturer, is not guaranteed or endorsed by the publisher.
