# Supplementary material for: Global public health implications of human exposure to viral contaminated water
Source: Front Microbiol. 2022 Aug 30;13:981896. doi: 10.3389/fmicb.2022.981896 (PMC9468673; doi:10.3389/fmicb.2022.981896)
Supplement: Supplementary file 1 [file Table_1.docx]

**Supplementary File: Public Health Implications of Human Exposure to Viral Contaminated Water**

Adedayo Ayodeji Lanrewaju^1^, Abimbola Motunrayo Enitan-Folami^1^*, Saheed Sabiu^1^, Joshua Nosa Edokpayi^2^, Feroz Mahomed Swalaha^1^

^1^Department of Biotechnology and Food Science, Durban University of Technology, P.O. Box 1334, Durban, 4000, South Africa

^2^Water and Environmental Management Research Group, Faculty of Science, Engineering and Agriculture, University of Venda, Thohoyandou, South Africa

*Correspondence:

Abimbola Motunrayo Enitan-Folami

[enitanabimbola@gmail.com](mailto:enitanabimbola@gmail.com)

**Keywords:** enteric viruses, wastewater, gastroenteritis, outbreak, wastewater-based epidemiology

**Supplementary Table 1**: Overview of the enteric viruses found in different water matrices, identification and quantification using advanced molecular techniques

| **Enteric virus** | **Genotype/Strain/Group** | **Method of detection** | **Water matrix** | **Country** | **Reference** |
| --- | --- | --- | --- | --- | --- |
| Human Adenovirus | HAdV 41 | ddPCR | SW (river) | France | Sedji et al., 2018 |
|  | ND | qPCR | Beach (seawater) | Brazil | Dias et al., 2018 |
|  | HAdV-C  HAdV-F | qPCR  ICC-qPCR nested-PCR | Coastal waters (beaches and lagoon) | Brazil | Staggemeier et al., 2017 |
|  | ND | RT-qPCR  ddPCR | WW (graywater, wastewater) | USA | Jahne et al., 2020 |
|  | ND | qPCR | SWG (raw & activated sludge) & WW (effluent) | Egypt | Elmahdy et al., 2020 |
|  | HAdV F 40 & 41 | qPCR | GW & WW (effluent) | Mexico | Rosiles‐González et al., 2019 |
|  | HAdV 41 & 12 | qPCR, nested-PCR | SW (river) & WW (raw & effluent) | Italy | Iaconelli et al., 2017a |
|  | HAdV 40 & 41 | Nested PCR | SW (lake) | Kenya | Opere et al., 2021 |
|  | HAdV 40 & 41 | PCR, Sequencing | WW & IW | Saudi Arabia | Nour et al., 2021 |
|  | ND | qPCR | SW (mangrove estuary) | Brazil | Keller et al., 2019 |
|  | ND | qPCR | SW (lake) | Louisiana | Cooksey et al., 2019 |
|  | ND | qPCR | WW (raw & effluent), SW (seawater & river) | Italy | Verani et al., 2019 |
|  | ND | qPCR | DW (raw and treated) | Canada | Sylvestre et al., 2021 |
|  | C-F | Nested-PCR, Sequencing | SW (stream) | Brazil | Girardi et al., 2018 |
|  | 41 | Nested-PCR, Sequencing | GW (spring park & hot spring) | Taiwan | Shih et al., 2017 |
| Rotavirus | Group A | RT-qPCR | SWG (raw & activated sludge) & WW (effluent) | Egypt | Elmahdy et al., 2020 |
|  | ND | qPCR | SW (beach & lagoon) | Brazil | Staggemeier et al., 2017 |
|  | Group A | qPCR | WW (effluent) | Brazil | Assis et al., 2018 |
|  | Group A | qPCR | SW (mangrove estuary) | Brazil | Keller et al., 2019 |
|  | ND | RT-qPCR | DW | Canada | Sylvestre et al., 2021 |
|  | ND | RT-qPCR | SW (sea) & DW (tap) | Italy | Purpari et al., 2019 |
|  | Group A | RT-PCR | SW (river) | Uruguay | Bortagaray et al., 2020 |
|  | Group A | RT-PCR | DW & SW | Japan | Miura et al., 2019 |
|  | G1P[8], G4P[8] & G9P[8] | qPCR | SW (river) | Argentina | Prez et al., 2020 |
|  | G2P[8], G3P[8], G9P[8],  G12P[8] | RT-qPCR, Semi-nested RT-PCR, Sequencing | SWG (WW) | Spain | Santiso-Bellón et al., 2020 |
|  | ND | RT-ddPCR | SWG (raw) | USA | Kiulia et al., 2021 |
|  | G1, G2, G3, G4, G8 & G9 | PCR | SW (dam and beach) | Argentina | Masachessi et al., 2018 |
|  | ND | RT-qPCR, Semi-Nested PCR | SW (Sea), WW (raw & effluent) & mixed waters | Italy | Cioffi et al., 2021 |
| Enterovirus | ND | qPCR | Coastal waters (beaches and lagoon) | Brazil | Staggemeier et al., 2017 |
|  | ND | Nested-PCR | SWG (raw & effluent) & SW (river) | Italy | Iaconelli et al., 2017a |
|  | Echovirus 3 | Nested-PCR, Sequencing | WW (effluent) | Italy | Iaconelli et al., 2017a |
|  | ND | RT-qPCR | DW | Canada | Sylvestre et al., 2021 |
|  | ND | RT-ddPCR | SWG (raw) | USA | Kiulia et al., 2021 |
|  | ND | RT-qPCR | SW (river) | China | Miao et al., 2018 |
|  | Enterovirus C99, Coxsackievirus A2, A13, A16, A22, B1, B3, B5, Echovirus 5, 6 & 9 | RT-qPCR, Cell culture, Sequencing | SWG (raw) & SW (Pond) | Uruguay | Lizasoain et al., 2018 |
|  | ND | Cell Culture | WW (effluent) | Iran | Moazeni et al., 2017 |
|  | ND | RT-qPCR | SW & DW | Portugal | Salvador et al., 2020 |
|  | ND | qPCR | DW | Nepal | Malla et al., 2019 |
| Norovirus | GII | Semi-nested PCR | DW & SW | Japan | Miura et al., 2019 |
|  | GI & GII | RT-qPCR  ddPCR | WW (raw) | USA | Jahne et al., 2020 |
|  | GI.2, GII.Pe, GII.P16 & GII.P17 | RT-qPCR | GW & WW (effluent) | Mexico | Rosiles‐González et al., 2019 |
|  | GI.2, GI.4, GI.6 & GII.1, GII.2 GII.4 | Nested-PCR | WW (raw & effluent) & SW (river) | Italy | Iaconelli et al., 2017 |
|  | GI & GII | RT-qPCR | WW (raw & effluent) & SW (river, estuarine & sea) | UK | Farkas et al., 2018 |
|  | GII | qPCR | SW (mangrove estuary) | Brazil | Keller et al., 2019 |
|  | GI & GII | RT-qPCR | DW | Canada | Sylvestre et al., 2021 |
|  | GII | RT-qPCR | SW (sea) & DW (tap) | Italy | Purpari et al., 2019 |
|  | GI.2, GI.4, GII.2, GII.6, GII.17 | RT-qPCR, Semi-nested RT-PCR, Sequencing | SWG (WW) | Spain | Santiso-Bellón et al., 2020 |
|  | GII | RT-qPCR | SW (river) | China | Miao et al., 2018 |
|  | GI & GII | qPCR | SW (dam & beach) | Argentina | Masachessi et al., 2018 |
|  | GI.1, GI.5, GII.P17 | RT-qPCR, Semi-Nested PCR Sequencing | SW (sea) WW (raw & effluent) & mixed waters | Italy | Cioffi et al., 2021 |
| Astrovirus | ND | RT-qPCR | SW (river) | China | Miao et al., 2018 |
|  | G1 - G7 | qPCR | SW (dam & beach) | Argentina | Masachessi et al., 2018 |
|  | CSstVa Type 1-7, MLB-AstV Type 1-3, VA-AstV Type 1-5 | RT-(semi-)nested-PCR, Next Generation Sequencing | WW (raw & effluent), GW & SW (river) | USA | Hata et al., 2018 |
|  | MAstV-1 | RT-qPCR, Semi-Nested PCR Sequencing | SW (sea & mixed waters) &WW (raw & effluent) | Italy | Cioffi et al., 2021 |
|  | 1, MLB1, MLB3 & BF34 | Next Generation Sequencing | SWG (raw) | China | Yang et al., 2021 |
|  | ND | PCR | WW (raw & effluent) | Kingdom of Bahrain | Janahi et al., 2020 |
|  | Genotypes 1 & 6 | RT-PCR, Sequencing | WW (effluent) | Tunisia | Ibrahim et al., 2017b |
|  | ND | qPCR | SW (reservoir & tributary) | Singapore | Goh et al., 2019 |
| Hepatitis A | ND | qPCR | SW (beach & sea) | Brazil | Dias et al., 2018 |
|  | IA & IB | Nested PCR | SWG (raw & effluents) & SW (river) | Italy | Iaconelli et al., 2017a |
|  | IA | qPCR | SW (dam & beach) | Argentina | Masachessi et al., 2018 |
|  | IB | Semi-Nested PCR | SWG (raw & effluent) | Iran | Nasiri et al., 2021 |
|  | ND | RT-qPCR | WW (raw) | USA | McCall et al., 2020 |
|  | ND | RT-qPCR | SW (river) & WW (effluent) | Kenya | Van Zyl et al., 2019 |
|  | ND | Nested RT-PCR | SW (river) | South Africa | Marie and Lin, 2017 |
| Hepatitis E | Genotype 3 | Nested-PCR | SWG (raw) & SW (river) | Italy | Iaconelli et al., 2017a |
|  | ND | Semi-nested RT-PCR | SW (sea) & DW (tap) | Italy | Purpari et al., 2019 |
|  | Genotype 3 | qPCR | SW (dam & beach) | Argentina | Masachessi et al., 2018 |
|  | Genotype 3 | RT-qPCR, Nested RT-PCR, Sequencing | WW (raw & effluent) | Germany | Beyer et al., 2020 |
|  | Genotype 3 | RT-qPCR, Nested RT-PCR & Automated Sequencing | SW (sea) | Italy | La Rosa et al., 2018 |
|  | Genotype 3 | NGS | DW (raw & tap water) | Sweden | Wang et al., 2020b |
| Aichivirus | ND | RT-qPCR, Semi-Nested PCR | SW (sea & mixed waters) & WW (raw & effluent) | Italy | Cioffi et al., 2021 |
|  | ND | Semi-Nested RT-PCR | SWG (raw, sludge & effluent), DNW & SW (river) | Egypt | Shaheen et al., 2020 |
|  | ND | qPCR | SW (reservoirs & tributaries) | Singapore | Goh et al., 2019 |
|  | B | RT-PCR, Direct Sequencing | WW | Tunisia | Ibrahim et al., 2017a |
|  | B | RT-PCR | SWG (WW) | South Africa | Onosi et al., 2019 |
|  | A | Illumina Miseq | SWG | Kenya | Hendriksen et al., 2019 |
| Coronavirus | SARS-CoV-2 | RT-qPCR | WW (raw & secondary effluent) | Spain | Randazzo et al., 2020 |
|  | SARS-CoV-2 | RT-qPCR, Nested-PCR | WW (secondary effluent) | Japan | Haramoto et al., 2020 |
|  | SARS-CoV-2 | RT-qPCR | WW (raw) & SW (river) | Italy | Rimoldi et al., 2020 |
|  | SARS-CoV-2 | RT-qPCR, Illumina Miseq | SWG (WW) | Australia | Ahmed et al., 2020 |

*PCR* Polymerase chain reaction, *qPCR* Quantitative PCR, *RT-PCR* Reverse transcription PCR, *RT-qPCR* Reverse transcription qPCR, *NGS* Next Generation Sequencing, *ND* not determined, *DNW* drainage water, *DW* drinking water, *GW* ground water, *IW* irrigation water, *SW* surface water, *SWG* sewage, *WW* wastewater
